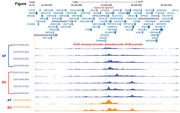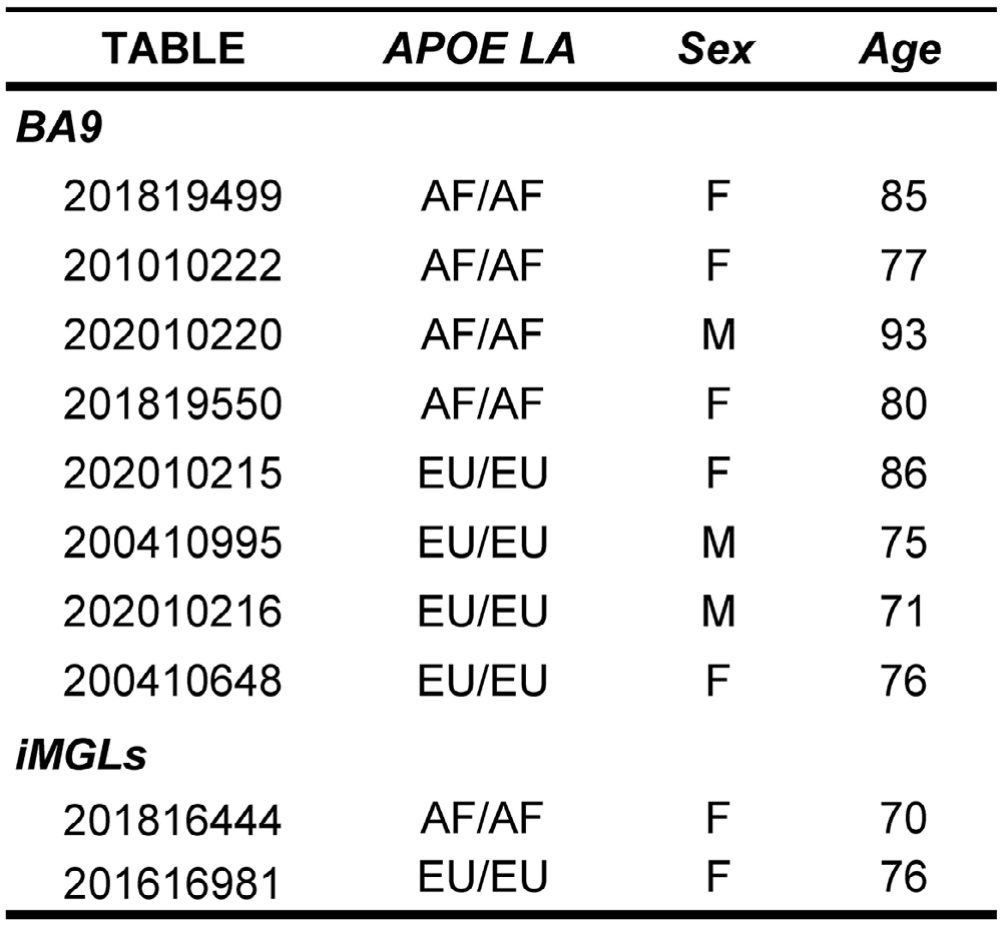# Enhanced Hi‐C Capture Analysis Revealed Differential Regulatory Architecture for APOE Between Ancestries

**DOI:** 10.1002/alz70855_106814

**Published:** 2025-12-25

**Authors:** Liyong Wang, Wanying Xu, Maria C Robayo, Luciana Bertholim Nasciben, Farid Rajabli, Sofia Moura, Aura M Ramirez, Marla Gearing, Sandra Weintraub, Changiz Geula, Theresa Schuck, David A. A. Bennett, William K. Scott, Larry D Adams, Katrina Celis, Derek M. Dykxhoorn, Karen Nuytemans, Margaret Pericak‐Vance, Anthony J Griswold, Juan I Young, Fulai Jin, Jeffery M Vance

**Affiliations:** ^1^ John P. Hussman Institute for Human Genomics, University of Miami Miller School of Medicine, Miami, FL, USA; ^2^ Department of Genetics and Genome Sciences, School of Medicine, Case Western Reserve University, Cleveland, OH, USA; ^3^ University of Miami Miller School of Medicine, Miami, FL, USA; ^4^ Goizueta Alzheimer's Disease Research Center, Emory University, Atlanta, GA, USA; ^5^ Mesulam Center for Cognitive Neurology & Alzheimer's Disease, Chicago, IL, USA; ^6^ Department of Pathology and Laboratory Medicine, Institute on Aging and Center for Neurodegenerative Disease Research, The Perelman School of Medicine at the University of Pennsylvania, Philadelphia, PA, USA; ^7^ Rush Alzheimer's Disease Center, Rush University Medical Center, Chicago, IL, USA

## Abstract

**Background:**

The APOEε4 allele is the major genetic risk factor for Alzheimer's disease (AD) but has different effect sizes across ancestral populations, with a larger effect in Non‐Hispanic Whites than African Americans. We have shown that 1) the local ancestry (LA) surrounding APOE is associated with higher genetic risk when of European (EU) rather than African (AF) ancestry (Rajabli et al, 2018); 2). APOE expression and chromatin accessibility are increased in brains with EU LA compared to AF LA (Griswold et al, 2021; Celis et al, 2023). Another important mechanism of cis‐transcriptional regulation is chromatin interactions. Herein, we examined the 3D genome structure at APOE on different ancestry backgrounds using Enhanced Hi‐C Capture Analysis (eHiCA).

**Method:**

Hi‐C analysis was done in frontal cortex and induced pluripotent stem cell (iPSC)‐derived microglia cells (iMGLs) from ε4/ε4 carriers with homozygous AF and EU LA at APOE. For each LA, four frontal cortex and one iMGL samples were examined (Table). HiCorr and DeepLoop were used to process Hi‐C data for enhanced chromatin interaction mapping and quantification. eHiCA was performed by using a 5 kb bait centered on the APOE promoter and extracting all chromatin interactions with the bait from a +/‐ 2 Mb of the surrounding genomic region.

**Result:**

In the frontal cortex, we observed ancestry‐biased chromatin interactions. In addition to a shorter‐range interaction between APOE promoter and a 50‐kb distant region, which is seen on both EU and AF LA, there are two longer‐range interactions between the APOE promoter and regions located 190 kb and 440 kb downstream of APOE, which are prominent on the EU LA but are suppressed on the AF LA. Similar ancestry‐biased chromatin interactions were observed in iMGLs.

**Conclusion:**

Compared to the AF genome, the EU genome has stronger and more chromatin interactions between APOE and other regions, correlating with its increased chromatin accessibility and providing a potential mechanism for the elevated APOE ε4 expression in EU brains. Ancestry‐biased chromatin interactions identified through eHiCA can guide functional analyses to understand the ancestry‐specific regulatory mechanisms in an effort to delineate the full AD regulatory landscape.